# The complete chloroplast genome of *Prunus phaeosticta* (Hance) Maxim. (Rosaceae) and its phylogenetic implications

**DOI:** 10.1080/23802359.2022.2163841

**Published:** 2023-01-15

**Authors:** Jia-Qi Wu, Yi Wang, Ping Sun, Zhong-Shuai Sun, Jian-Sheng Shen

**Affiliations:** a Flower, Forestry and Fruit Institute, Jinhua Academy of Agricultural Sciences, Jinhua, China; b Zhejiang Provincial Key Laboratory of Plant Evolutionary Ecology and Conservation, Taizhou University, Taizhou, China

**Keywords:** *Prunus phaeosticta*, Laurocerasus, chloroplast genome, phylogenomics

## Abstract

The complete chloroplast (cp) genome of *Prunus phaeosticta* (Hance) Maxim. has been characterized by reference-based assembly using Illumina paired-end data. The circular complete cp genome is 158,752 bp in length, comprising a large single-copy (LSC) region of 87,085 bp, a small single-copy (SSC) region of 18,923 bp, and a pair of inverted repeats (IRs) of 26,372 bp.A total of 129 functional genes were identified, including 84 protein-coding genes, 37 tRNA genes, and 8 ribosomal RNA genes. The phylogenetic analysis showed that *P. phaeosticta* displayed a kinship to *Prunus zippeliana*.

## 1. Introduction

*Prunus* L. (*Prunus sensu lato*) is well known for its taxonomic complexity, the phylogenetic relationships among subgenera, sections, and species are still unclear (Wen et al. 2008; Shi et al. 2013; Chin et al. 2014). The most recent classification of the genus recognized only three subgenera: *Prunus*, *Cerasus* (Mill.) A. Gray, and *Padus* (Mill.) Peterm., with a broader concept of subg. *Padus* that included *Laurocerasus* Duhamel and the former genera *Maddenia* Hook. f. & Thoms and *Pygeum* Gaertn. (Shi et al. 2013). Recent phylogenetic studies revealed *Prunus* subg. *Padus* to be polyphyletic, taxa of subg. *Padus* and subg. *Laurocerasus* are highly intermixed in phylogenetic trees reconstructed using a limited set of short DNA sequence (Liu et al. 2013; Chin et al. 2014; Zhao et al. 2016). The chloroplast genome is a robust and appropriate tool that could provide much better ability than the universal DNA markers on revealing phylogeny and evolutionary history of plants (Gitzendanner et al. 2018; Do et al. 2020; Su et al. 2021) However, genomic resources of *Laurocerasus* species are extremely scarce (Zou et al. 2019). In order to better understand the molecular phylogenetic relationship among the subg. *Padus*, we assembled and characterized the first complete chloroplast genome of *Prunus phaeosticta* (Hance) Maxim. 1883 using the next-generation sequencing technology. Since *P. phaeosticta* is one of the most widespread species, which ranges from India and Thailand all the way to East China and contains seven forms, we chose to sequence it to contribute genetic resources and enhance understanding of this polymorphic species. Furthermore, a phylogenomic analysis of 14 *Prunus* species was also presented. The results will lay a solid foundation for the future phylogenetic researches of *P. sensu lato*.

## 2. Materials and methods

### 2.1. Plant materials and DNA extraction

**Figure 1.**
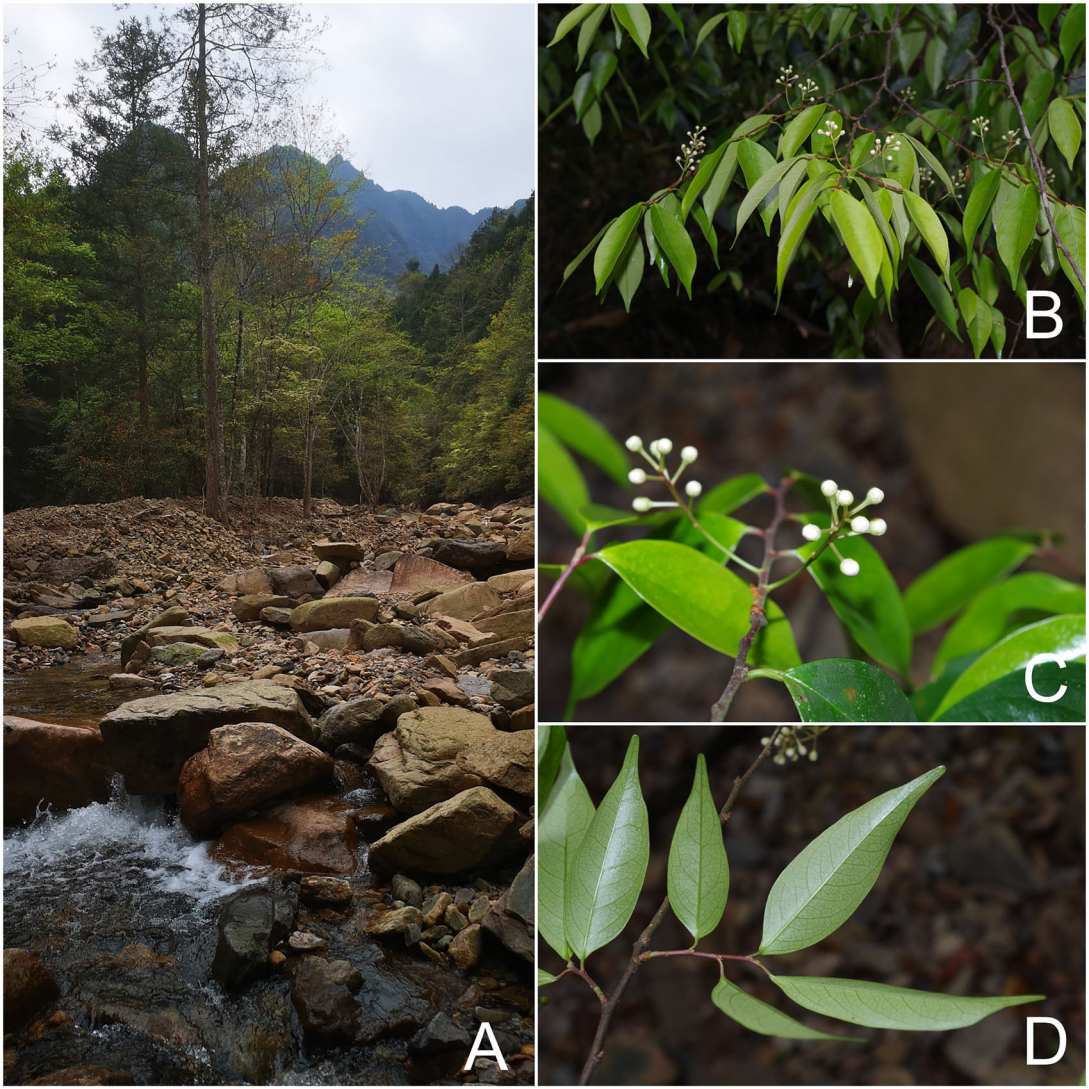
Pictures of *P. phaeosticta* taken on April 14th 2020, at Mt. Jiulong, Suichang County, Zhejiang Province, China. (A) Natural habitat; (B) flowering branches; (C) inflorescences and leaves; (D) abaxial side of leaves.

Fresh young leaves of *P. phaeosticta* (Figure 1) were collected from Mt. Jiulong, Suichang County, Zhejiang province, China (28.3850°N, 118.8927°E). A voucher specimen and its DNA sample were deposited at the Herbarium of Zhejiang University (HZU, https://www.zju.edu.cn/; collector: Pan Li, panli_zju@126.com) under the voucher number LP208067. Genomic DNA was extracted using a modified CTAB protocol (Doyle and Doyle 1987).

### 2.2. Plastome sequencing, assembly, and annotation

The next-generation sequencing was performed with an Illumina NovaSeq platform (Illumina, San Diego, CA). The cp genome was assembled *via* NOVOPlasty 2.6.3 (Dierckxsens et al. 2017), using *Prunus zippeliana* (NC043926, Zou et al. 2019) as reference genome. Genome annotation was conducted using the online software GeSeq (Tillich et al. 2017) by comparing with the *P. zippeliana* cp genome (NC043926, Zou et al. 2019). Geneious R11 (Biomatters Ltd., Auckland, New Zealand) was used for inspecting the genome structure. The circular gene map of was drawn by the cpgavas2 web server (http://47.96.249.172:16019/analyzer/view).

### 2.3. Phylogenetic analysis

To reveal the phylogenetic position of *P. phaeosticta*, a phylogenetic analysis intra subg. *Padus* was evaluated based on protein coding regions (CDS) of cp genome of *P. phaeosticta* and 13 reported cp genomes from *Prunus* (NC054253, Li et al. 2021; NC035891, Xu et al. 2018; NC043926, Zou et al. 2019; NC026982, NC036133, Luan et al. 2018.; MW801161 Jin et al. 2021; NC059022, NC059023, NC059024, NC059025, NC059026, NC059027, Su et al. 2021; NC053881, Wang et al. 2021). Eighty-four shared CDS were extracted and aligned by MAFFT (Katoh et al. 2005). Prunus *fasciculata* (NC054253) and *P*runus *cerasoides* (NC035891) were selected as outgroup. We reconstructed a phylogeny employing the GTR + G model and 1000 bootstrap replicates under the maximum likelihood (ML) inference in RAxML-HPC v.8.2.10 on the CIPRES cluster (Miller et al. 2010).

## 3. Results

### 3.1. Genome organization and compositions

**Figure 2.**
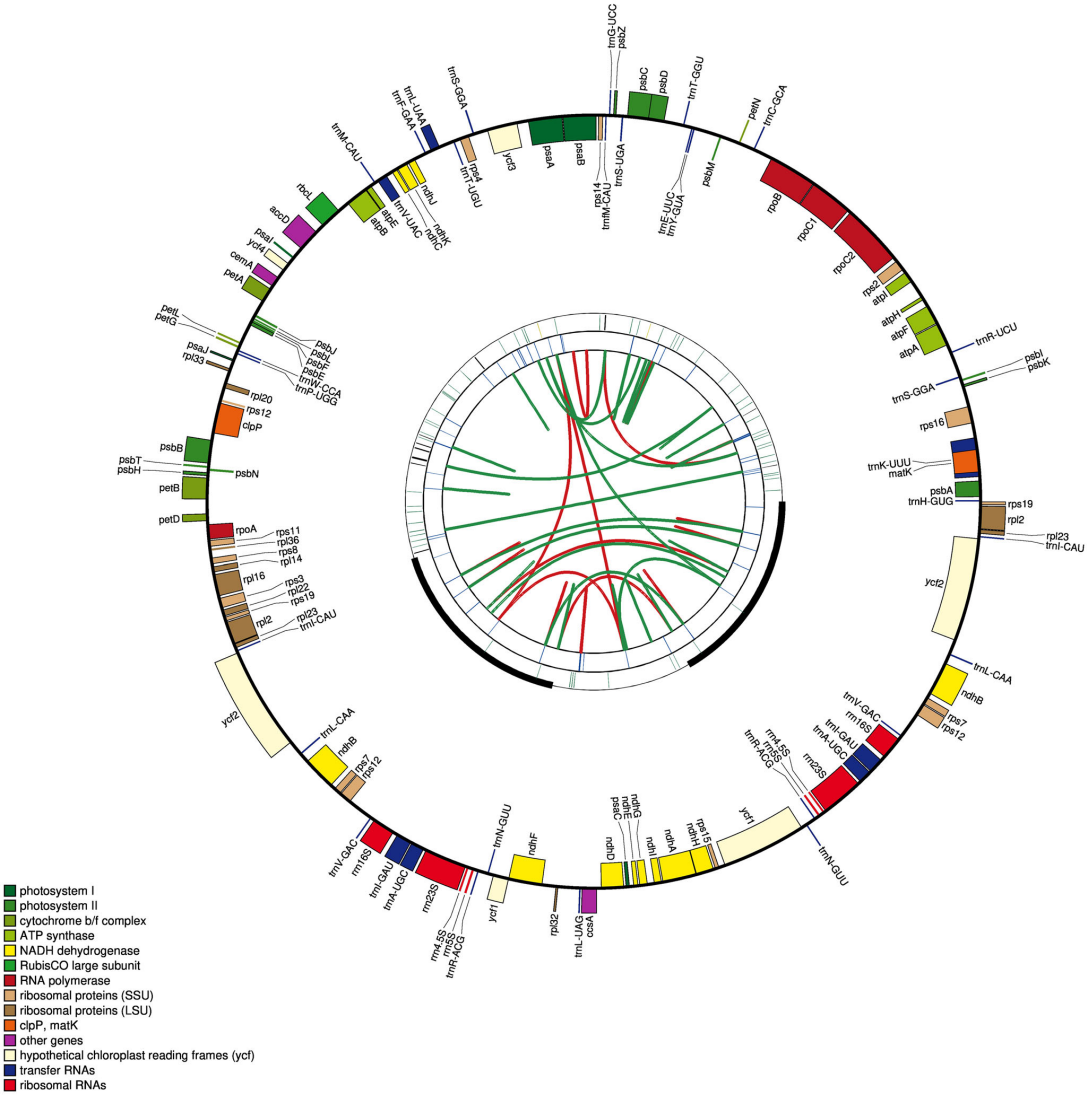
Graphic representation of features identified in *P. phaeosticta* chloroplast genome using CPGAVAS2. The functional classification is shown at the bottom left.

The chloroplast genome of *P. phaeosticta* presented a typical circular DNA molecule with a total length of 158,752 bp (Figure 2). It has a characteristic quadripartite structure with a large single-copy (LSC) region of 87,085 bp, a small single-copy (SSC) region of 18,923 bp, and a pair of inverted repeats (IRs) of 26,372 bp. The total GC content in the chloroplast genome of *P. phaeosticta* was 38.6%. The GC content of the IR region (43.4%) was higher than those of the LSC (37.0%) and SSC (33.4%) regions.

A total of 129 functional genes were identified, including 84 protein-coding genes (PCGs), 37 tRNA genes, and 8 rRNA genes. Of these genes, 15 genes (rps16, atpF, rpoC1, petB, petD, rpl16, rpl2, ndhB, ndhA, trnK-UUU, trnG-GCC, trnL-UAA, trnV-UAC, trnI-GAU, and trnA-UGC) had one intron and 3 genes (rps12, clpP, and ycf3) had two introns. Most genes occurred in a single copy, while six PCGs (rpl2, rpl23, ycf2, ndhB, rps7, and rps12), seven tRNA genes (trnI-CAU, trnI-GAU, trnL-CAA, trnV-GAC, trnA-UGC, trnR-ACG, and trnN-GUU), and four rRNA genes (rrn4.5, rrn5, rrn16, and rrn23) in IR regions are duplicated.

### 3.2. Phylogenetic analysis

**Figure 3.**
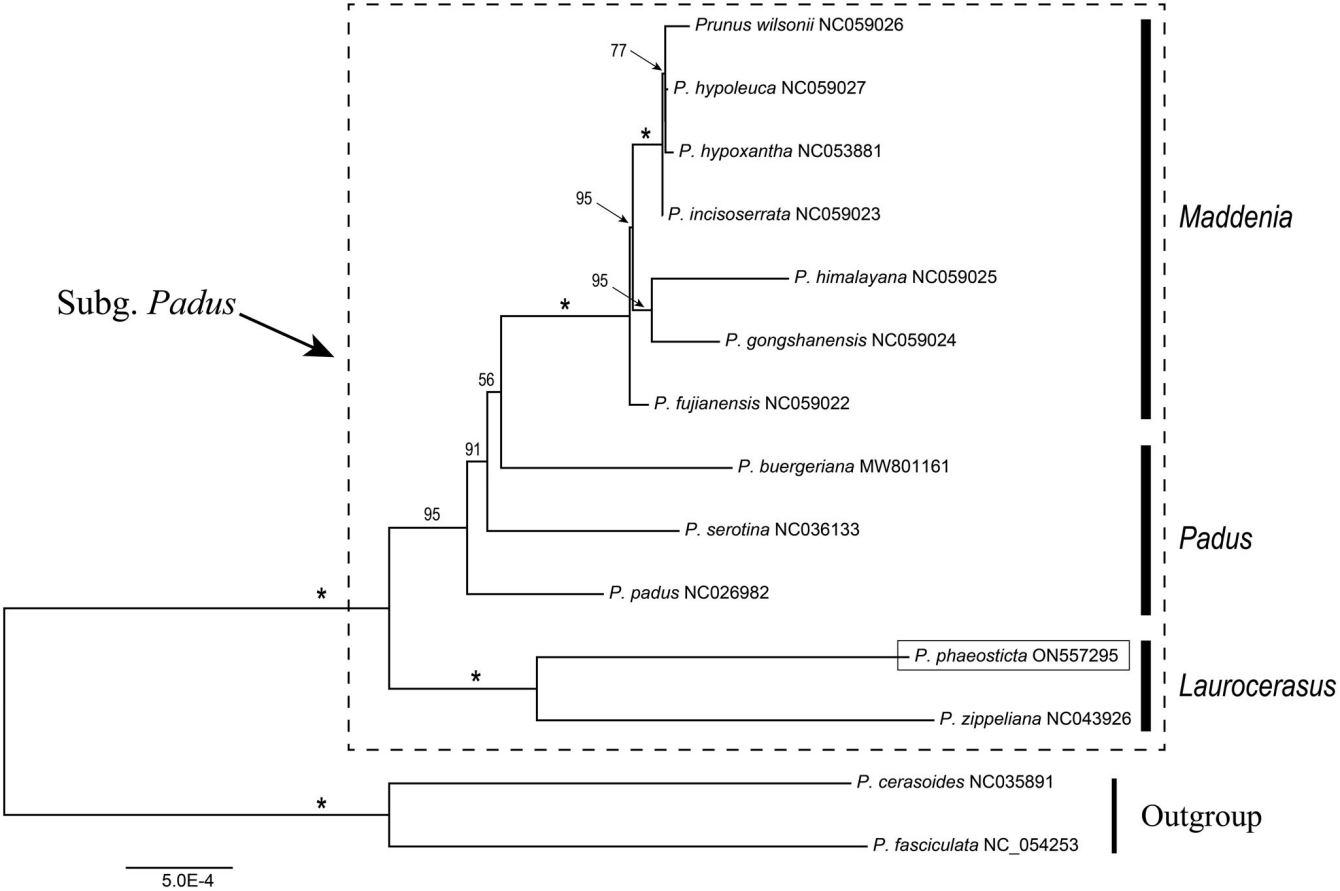
Maximum likelihood (ML) tree reconstruction of 14 taxa from *Prunus* based on 84 shared CDS in the chloroplast genomes. Relative branch lengths are indicated. Support values above the branches are ML bootstrap support; ‘*’ indicates 100% support values.

A robust phylogeny of subg. *Padus* was obtained based on the CDS data (Figure 3). In our phylogenetic tree, the 14 species were clustered into two main clades: 1) *Laurocerasus* clade; 2) *Maddenia* & *Padus* clade. *Maddenia* species formed a monophyletic clade, but *Padus* species were revealed as a paraphyletic group (Figure 3). According to the phylogenetic tree, *P. phaeosticta* and *P. zippeliana* clustered within the *Laurocerasus* clade and the sister relationships between *P. phaeosticta* and *P. zippeliana* were observed.

## 4. Discussion

As described previously, *Prunus* L. (*P. sensu lato*) is well known for its taxonomic complexity. Although some comprehensive studies on the phylogenetic relationships of *P. sensu lato* has significantly promoted the understanding of the interspecies relationships in this genus (Wen et al. 2008; Liu et al. 2013; Shi et al. 2013; Chin et al. 2014; Zhao et al. 2016), but limited data of genome level has become an impediment to resolve the phylogeny and the interspecies relationships of *P. sensu lato.* In this study, we sequenced the chloroplast genome of *P. phaeosticta* and conducted a systematic analysis of the chloroplast genomes with 13 other *Prunus* chloroplast genomes. Our systematic analysis result was consistent with the recent phylogenetic study on *P. sensu lato*, the monophyly of *Maddenia* was supported and the *Padus* species were paraphyletic (Shi et al. 2013; Chin et al. 2014; Su et al. 2021). According to our result, *P. phaeosticta* clustered within the *Laurocerasus* group and exhibited the closest relationship with *P. zippeliana*. We expect that the cp genome of *P. phaeosticta* will be a valuable genomic resource for future taxonomy, phylogeny studies on *P. sensu lato* and new *Prunus* cultivar breeding.

## Data Availability

The genome sequence data that support the findings of this study are openly available in GenBank of NCBI at (https://www.ncbi.nlm.nih.gov/) under the accession no. ON557295. The associated BioProject, SRA, and Bio-Sample numbers are PRJNA841240, SRR19348248 and SAMN28578094, respectively.
